# Health Economic Evaluations of Visceral Leishmaniasis Treatments: A Systematic Review

**DOI:** 10.1371/journal.pntd.0003527

**Published:** 2015-02-27

**Authors:** Daniel S. Marinho, Carmen N. P. R. Casas, Claudia C. de A. Pereira, Iuri C. Leite

**Affiliations:** 1 Centro de Desenvolvimento Tecnológico em Saúde, Fundação Oswaldo Cruz, Rio de Janeiro, Brasil; 2 Escola Nacional de Saúde Pública Sérgio Arouca, Fundação Oswaldo Cruz, Rio de Janeiro, Brasil; 3 National Institute for Science and Technology on Innovation on Neglected Diseases (INCT/IDN), Fundação Oswaldo Cruz, Rio de Janeiro, Brasil; Johns Hopkins Bloomberg School of Public Health, UNITED STATES

## Abstract

Visceral leishmaniasis (VL) is a severe form of the leishmaniasis-disease complex. Its importance to public health relies on its high fatality rate in non-treated cases, the socio-economic impact related to its morbidity, and its endemicity on different continents. The estimated burden of disease of VL varies from 1,969,000 to 2,357,000 Disability Adjusted Life Years (DALYs). VL is classified as a Neglected Tropical Disease (NTD), and is strongly related to poverty and its consequences. Visceral leishmaniasis calls for the development of cost-effective technologies for diagnosis and treatment.

**Objective:**

The main objective of this study was to identify, describe, classify and analyze the scientific health economic evidence of VL-related technologies.

**Methods:**

A web search of combinations of free text and Mesh terms related to the economic evaluation of visceral leishmaniasis was conducted on scientific publication databases (Web of Science, Scopus, Medline via the Pubmed and Lilacs). A manual search of references lists of articles previously identified by the authors was also included. Articles written in English, Portuguese, Spanish or French were considered suitable for inclusion. Articles that matched the inclusion criteria were screened by at least two researchers, who extracted information regarding the epidemiologic scenario and methodological issues on a standardized form.

**Results:**

The initial search retrieved 107 articles, whose abstracts were inspected according to the inclusion criteria leading to a first selection of 49 (46%) articles. After the elimination of duplicates, the list was reduced to 21 (20%) articles. After careful reading and application of exclusion criteria, 14 papers were eligible according to the description, classification and analysis process proposed by the study. When classified by type of economic evaluation, articles were 7 (50%) cost-effectiveness, 5 (36%) cost-minimization, 1(7%) cost-benefit, and 1(7%) budget impact. When classified by methodology, studies were mainly nested to clinical-trials (“piggy back”) 8(57%). Discount rates for outcomes and costs were present in 3 (43%) of the cost-effectiveness studies, and according to WHO's recommendations, the discount rate of 3% was used in all studies.

**Conclusions:**

This article showed that health economic evaluations on visceral leishmaniasis used a wide range of technologies and methods. Nevertheless it is important to point out the geographic concentration of studies, which makes their transferability uncertain to different epidemiological scenarios, especially those concerning visceral leishmaniasis caused by *Leishmania infantum*.

## Author Summary

Visceral leishmaniasis (VL), also known as kala azar, is a neglected tropical disease caused by parasitic protozoa of the genus *Leishmania*. VL is related to poverty and its consequences, which leads to its status of neglected disease. For that reason, cost-effective forms of diagnoses and treatment are very important and still needed. This research aimed at a better understanding of the publications about the technologies currently available, from the standpoint of their economic value. For that purpose, we conducted a systematic review of the literature in order to identify the papers that conducted economic evaluations of technologies used in VL. We initially retrieved 107 articles, which were inspected according to specific guidelines for systematic reviews. After that process, 14 articles matched the inclusion criteria in our review. We classified those studies according to the type of economic evaluation they made, and the methodology used in each one. We found evaluations about a variety of technologies, but the studies were geographically concentrated in Asia, more specifically in India. This concentration is not good because the disease also affects other continents and it is not possible to transfer the economic evaluation from one country or epidemiologic scenario to another.

## Introduction

During past decades, health economic evaluations have become increasingly important to the evaluation of new health technologies. Many countries have addressed common issues related to the process of health technology assessment (HTA) while elaborating guidelines regarding the process to evaluate such technologies, and the evidence-based decision making has been adopted by health systems around the world and by academia[1,2] Nevertheless, health technology assessment and specifically health economic evaluation are still scarce for the so-called neglected tropical diseases (NTD). This is an alarming fact, considering that NTDs present higher burden of disease than some non-communicable diseases[3,4], and mainly affect the poorest regions and populations of the globe[5–7], to the point of being classified as diseases of poverty. For that reason, supporting an efficient resource allocation process with health economic evidence is imperative, as poor populations are vulnerable to a wide spectrum of diseases, and are assisted by budget restricted health systems.

Visceral leishmaniasis (VL), one of the NTDs, is a life threatening infectious disease affecting around 500,000 and killing 50,000 individuals a year[8–10]. India, Brazil, Sudan, Nepal, Ethiopia and Bangladesh concentrate 90% of cases[8]. Malaria is currently the sole tropical infectious disease responsible for more deaths than VL. Notwithstanding the public health importance of VL, the availability of new treatments is very restricted and the narrow development pipeline of new strategies to manage this disease (control, diagnostic, treatment) highlights the importance of evaluating the technologies already on the market and of forecasting the scenarios of new technology incorporation.

The objective of this review was to identify, describe and classify the scientific production on economic evaluations of VL interventions. We adopted a similar methodology to the study by Walker and Fox-Rushby[11] for communicable diseases in developing countries.

## Methods

### Search Strategy

A web search combining free text and MeSH terms related to the economic evaluation of Visceral Leishmaniasis was used in Web of Science, Scopus, Medline via the Pubmed and Lilacs (Table 1), without language or publication date restriction. The search was carried out during January of 2013. A manual search of references lists of articles previously identified by the authors was also included, although the web search was able to cover the entire reference list identified in articles. The aim of the search strategy was to identify economic evaluation studies. An economic evaluation encompasses the study of costs and outcomes related to the use of a technology. The simplest form of evaluation is a cost-minimization analysis, which consists of comparing only the costs related to the use of a new technology compared to an alternative called the compactor. This approach assumes that there is no difference in the outcomes, so the goal is to identify the least costly option. Another type of economic evaluation is called cost-effectiveness analysis in which a comparison of the various technology options is undertaken, and the costs are measured in monetary units, and then aggregated, and outcomes are expressed in natural (non-monetary) units, which are called effectiveness of the technology. A similar kind of economic evaluation consists of comparing various options, in which costs are also measured in monetary units and outcomes are measured in a utility units, usually quality-adjusted life years or disability-adjusted life years. One can also conduct an evaluation of the financial impact of the introduction of a technology on the budgets of a government or agency.

**Table 1:** Search strategy.

BASE	SEARCH KEY	RESULTS	SELECTED
Web of Science	TS = ("cost-effectiveness" OR "cost-utility" OR "cost-minimization" OR "cost-benefit" OR "economic impact") AND TI = "visceral leishmaniasis"	18	16
PUBMED	(("cost-effectiveness" OR "cost-utility" OR "cost-minimization" OR "cost-benefit" OR "economic impact"[Title/Abstract])) AND "visceral leishmaniasis"[Title]	22	18
SCOPUS	TITLE-ABS-KEY("cost-effectiveness" OR "cost-utility" OR "cost-minimization" OR "cost-benefit" OR "economic impact") AND TITLE("visceral leishmaniasis")	66	14
LILACS	“costo efectividad” [palavras] and “leishmaniasis” [palavras]	1	1

### Inclusion Criteria

The study included all articles that explicitly proposed to conduct economic evaluations on visceral leishmaniasis in the title, abstract or objectives. Articles written in English, Portuguese, Spanish or French were considered suitable for inclusion.

### Exclusion Criteria

After analyses by two researchers, studies that did not describe economic evaluations, or did not analyze interventions to control visceral leishmaniasis, editorials, reviews or methodological articles were excluded.

### Data Analysis

All articles selected were evaluated and data were organized on a standardized spreadsheet prepared to collect relevant information on articles, definitions, methods and results. Articles were evaluated considering the country of origin; the payers perspective; study design; technologies evaluated (table 2); comparator; cost-effectiveness threshold adoption; outcomes measures.

**Table 2:** Technologies evaluated.

ID	Transmission Control			Vaccine	Diagnostic Tools			Drugs					
	Insecticide	Dog Collar	Dog Culling		DAT	RK39	Parasitology	Antimoniate	Amphotericin B Deoxiclate	Amphotericin B Lipidic complex	Amphotericin BLipossomal	Miltefosin	Paramomycin
A	*					*		*	*			*	
B	*		*		*		*	*					
C								*					
D					*		*	*					
E	*												
F								*					
G				*									
H								*	*		*	*	*
I									*				
J											*	*	*
K								*					
L						*							
M								*		*			
N								*	*		*	*	

Protocol registration: The current systematic review protocol was registered on the International prospective registry of systematic reviews—PROSPERO and received registration id: 2014:CRD42014007534

## Results

### Search Strategy

**Fig 1:**
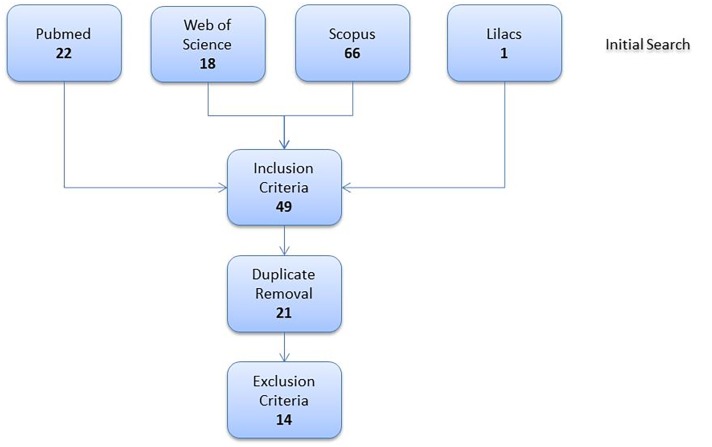
Selection of articles.

The initial search retrieved 107 articles whose abstracts were screened for inclusion criteria, leading to 49 abstracts that were also screened for duplicates. This strategy generated a list of 21 articles that were evaluated according to defined exclusion criteria by at least two researchers, thus resulting in 14 articles[12–25] included for description and analysis, Fig 1.

### Characteristics of Studies

**Fig 2:**
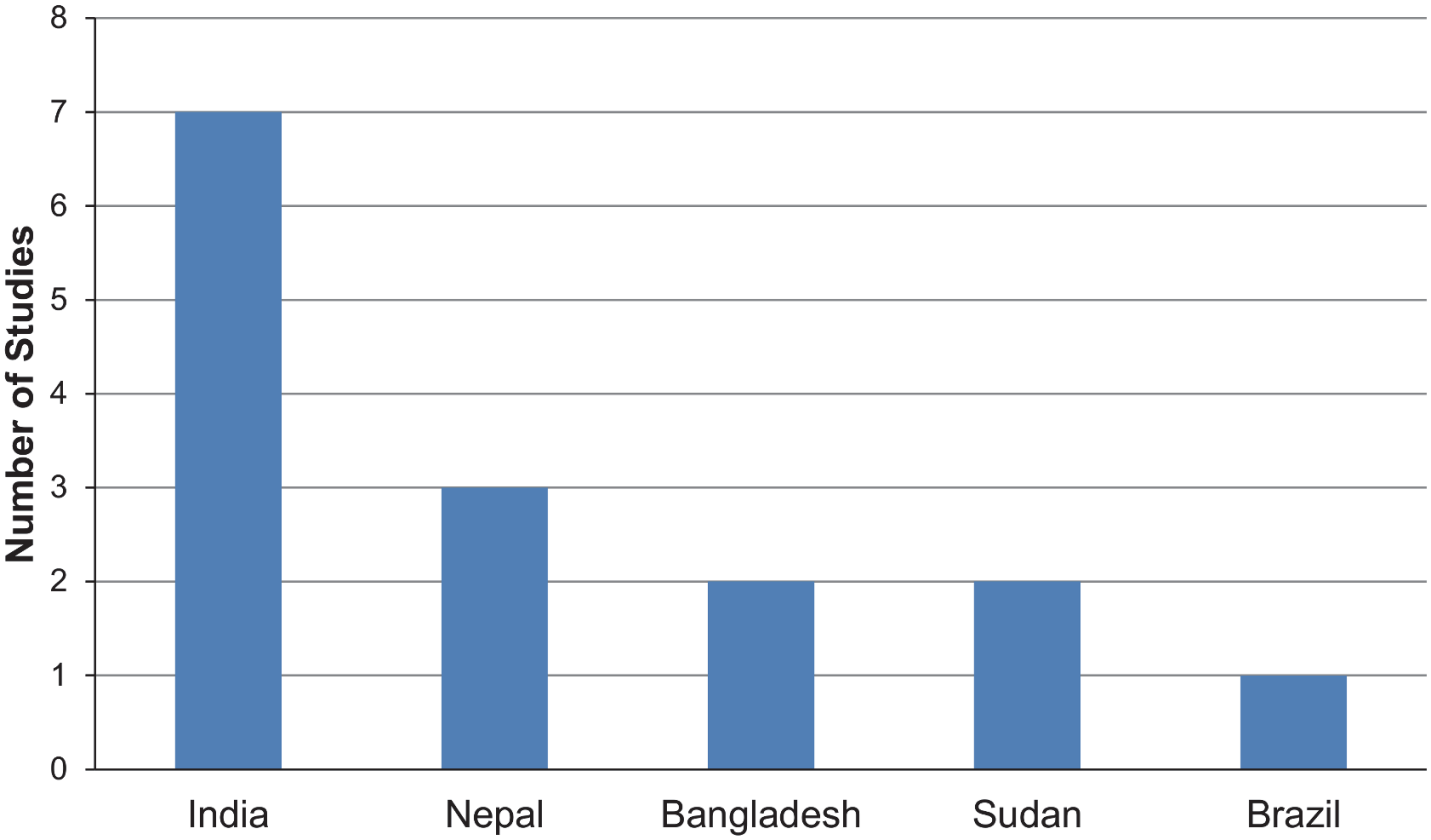
Geographic distribution of studies.

The analysis regarding country of origin of the 14 studies selected, Fig 2, showed a concentration of studies in the same five countries responsible for 90% of LV cases. We should highlight that India concentrates almost 7(50%) of studies and that, regardless of all its specificities, VL in the Americas accounts for only 1(7%) article published in 1996.

All articles were published from 1996 to 2012 based on data from 1988 to 2011, Fig 3.

**Fig 3:**
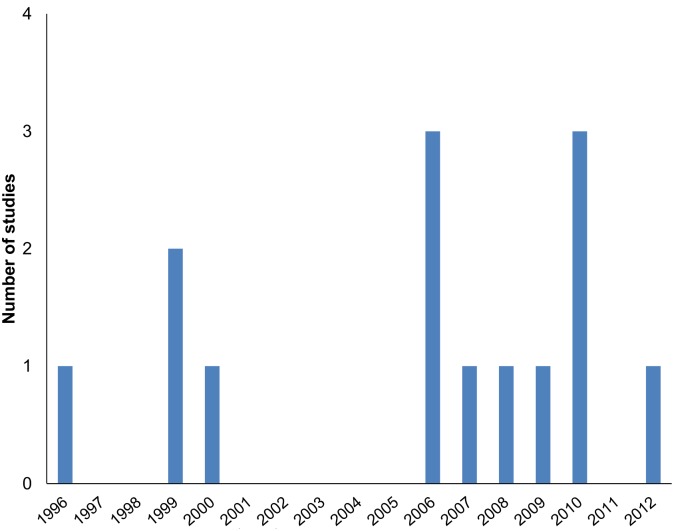
Time distribution of studies.

**Fig 4:**
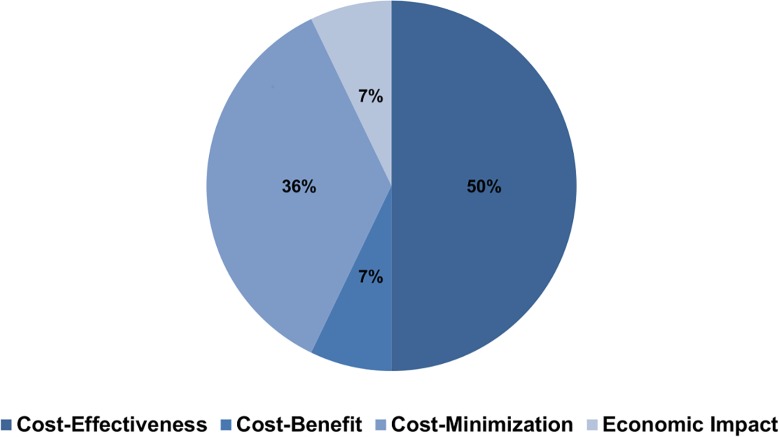
Studies by type of economic evaluation.

**Fig 5:**
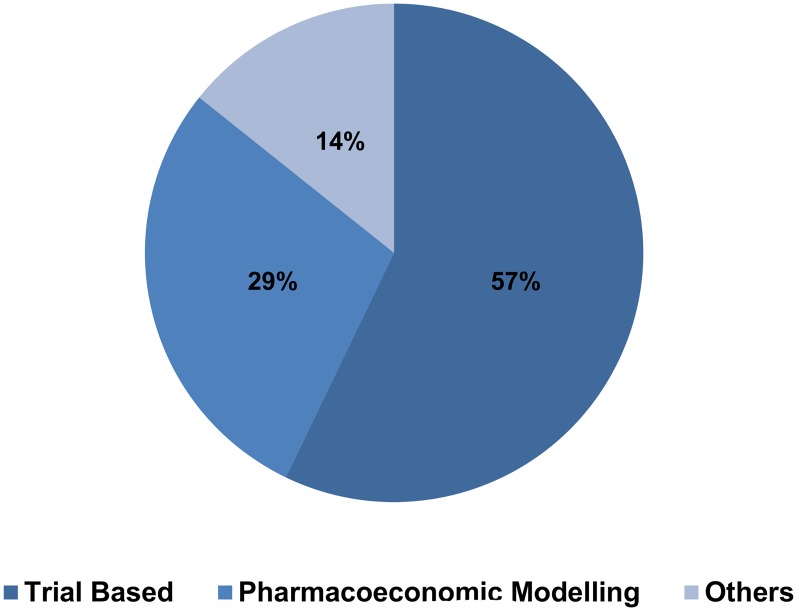
Economic evaluations by method used.

Articles were classified by type of economic study, whether cost-effectiveness, cost-benefit, cost-minimization or cost-of-illness analysis. Considering this classification, cost-effectiveness studies were the most frequent, representing 7(50%) of total studies Fig 4. Technologies assessed are presented in Table 2, and as expected they mainly refer to drugs, albeit the small number of studies evaluating the most recent drugs available. This pattern was also present in the evaluation of diagnostic methods with only two articles assessing the new rapid test for VL diagnosis. It is also important to point out that technologies aiming at the transmission of VL are very under assessed in the literature reviewed, capturing only one cost-of-illness article that evaluated the use of insecticides, and one cost-effectiveness analysis of vaccines that was only possible by the simulation of scenarios regarding the hypothetical intervention. In relation to methods applied, studies were classified in the following categories: clinical-trials (piggy-back)[26], pharmacoeconomic modelling, and others (i.e.: surveys). It is important to point out that 8(57%) of economic evaluations of VL were trial based Fig 5.

Among the 13 (93%) articles that declared the perspective of the analysis, 5 (38%) adopted the societal perspective and 8 (62%) performed economical evaluation according to the payers’ perspective, 2(25%) of which with patients or their families considered as payers, and 6 (75%) to the health system. Outcomes were presented as averted Years of Life Lost, 1 (7%); patients cured, 1 (7%); monetary units, 2 (14%); averted deaths, 3 (21%); averted disability-adjusted life years (DALY), 3 (21%); and “treatments”, 4 (28%). Most studies presented monetary units as US$, 13 (93%); and one article presented Nepalese Rupies NR, 1 (7%). Cost-effectiveness threshold was presented by 4 (57%) of the CE studies; three (75%) of these studies adopted the WHO choice criteria of 1 to 3 x GDP per capita/ averted DALY[27]; and 1 (25%) study adopted a threshold of U$ 25/ averted DALY. It is important to note that only one study presented a CE acceptability curve. The only three (43%) CE studies that applied discount rates to cost or outcomes chose a rate of 3% a year.

Sensitivity analyses were performed on 7 (50%) studies, 72% of which used multivariate sensitivity analyses. Modelling was used by 29% of all selected studies, with three (75%) based on decision trees and one (25%) based on Markov chain models.

Table 3 presents the outcomes, and inflation adjusted costs for the most cost-effective (or cost-saving) intervention of each study. The inflation adjusted costs were also purchasing power parity converted considering the 2013 PPP conversion rate[28].

**Table 3:** Costs and outcomes for the most cost-effective (cost saving) interventions.

Study ID	Costs I$	Outcome measure
A	0.014	1 I$
J	8.4024	Averted YLL
E	21.9891	Household
F	238.9442	Averted DALY
B	267.6986	Averted DALY
H	382.3464	Averted death
C	391.1861	Averted death
L	603.0658	Treated patient
K	611.7021	Treated patient
N	1733.6182	Averted death
M	1887.9591	Treated patient
D	2316.0403	Averted death
I	2834.4347	VL case
G	2861.9950	Averted DALY

All articles were evaluated following the Consolidated Health Economic evaluation reporting standards (CHEERS) statement[29], and the result is presented as supplementary material. It’s interesting to note that the majority of studies lacked information on time horizon 12(85,7%); measurement of effectiveness 12(85,7%); measurement and valuation of preference based outcomes 13(92,9%); characterizing uncertainty 12(85,7%); characterizing heterogeneity 12(85,7%); and conflicts of interest 12(85,7%).

## Discussion

Despite the importance of VL as a public health problem[30], expressed by its estimated world burden of disease of 2,357,000[31] disability-adjusted life years, the search strategy was not able to identify a large number of articles on VL health economic evaluation. This fact may be explained in part by the scarce development of VL control strategies during past years, but it may also reflect the unawareness of poverty related diseases.

The analysis of the articles selected showed a large concentration of studies carried out in the Asian continent, especially in India, which is the country with the highest VL incidence[32]. Due to such concentration, the literature has not dealt with some local VL characteristics such as: different Leishmania species causing VL; different vector species, specific site related hosts; heterogeneous drug resistance profiles; and local socio-demographic characteristics. As a complex vector transmitted disease, VL control is not a straightforward action. It relies on interventions to control transmission (including vaccines), diagnosis, and treatment [8,10]. By disregarding these characteristics, most of the studies did not consider the assessment of combined interventions, what may be a weakness for the economic evaluation of transmissible diseases.

Although the scientific literature has previously emphasized the advantages of using dynamic models while conducting cost-effectiveness studies on infectious diseases[26,33], none of the studies analyzed was based on dynamic transmission models. This fact may reflect an excessive simplification of VL disease history, by not including the importance of interactions of hosts and vectors, or by not dealing with the development of drug resistance.

When presenting cost-effectiveness acceptability thresholds, the authors decided to follow WHO recommendations of less than 3 x GDP per QALY or averted DALY for cost-effective interventions and less than 1 x GDP per QALY or averted DALY for very cost-effective interventions. Nevertheless, it is very important to mention that these thresholds are not able to address income inequality, which is particularly high in endemic countries of visceral leishmaniasis.

Despite the representation of endemic countries on the investigation teams, the majority of articles were produced with foreign cooperation, suggesting that the capillarity of the techniques used in economic evaluations of health interventions are still a challenge for developing countries.

### Conclusion

Visceral leishmaniasis is still an important infectious disease in many countries, especially in developing ones, so its control is of great public health importance. The development of new technologies is imperative in order to properly address VL in order to control epidemics and reduce its impact on society[34].

The present review has showed that health-economic studies, which are an essential part of the health technology assessment and incorporation process, were not able to overcome gaps in knowledge of strategies to deal with such a debilitating disease.

It is also important to underscore that the majority of studies accessed by this article did not consider the societal perspective to guide the evaluation; they mainly adopted the payers' perspective, which does not necessarily express the entire dimension of the health intervention evaluated.

Transmission control was only assessed by three studies, which may reflect the difficulty of evaluating these strategies due to the interval between intervention and epidemiologic impact, or the difficulty of linking intervention and impact.

Despite the representation of endemic countries on the investigation teams, the majority of articles were produced with foreign cooperation, suggesting that the capillarity of the techniques used in economic evaluations of health interventions are still a challenge for developing countries.

Most recent treatments for VL (ex. miltefosine, and paramomycin) were evaluated only a few times, and should be evaluated in different epidemiological scenarios. Future studies should consider a longer time horizon, so that the infectious disease characteristics and peculiarities of visceral leishmaniasis could be better expressed and accounted for.

## Supporting Information

S1 CHEERS Evaluation (XLSX)

S1 PRISMA Evaluation (DOC)

## References

[1] ISPOR global health care systems road map (n.d.). International Society of Pharmacoenomics and Outcomes Research. Available: http://www.ispor.org/HTARoadMaps/Default.asp.

[2] INAHTA—Home (n.d.). Available: http://www.inahta.org/. Accessed 6 May 2008.

[3] GBD Arrow Diagram | Institute for Health Metrics and Evaluation (n.d.). Available: http://www.healthdata.org/data-visualization/gbd-arrow-diagram. Accessed 7 January 2015.

[4] Hotez PJ, Alvarado M, Basáñez M-G, Bolliger I, Bourne R, et al (2014) The Global Burden of Disease Study 2010: Interpretation and Implications for the Neglected Tropical Diseases. PLoS Neglected Tropical Diseases 8: e2865 10.1371/journal.pntd.0002865 25058013 PMC4109880

[5] Boelaert M, Meheus F, Sanchez A, Singh SP, Vanlerberghe V, et al (2009) The poorest of the poor: a poverty appraisal of households affected by visceral leishmaniasis in Bihar, India. Trop Med Int Health 14: 639–644. 10.1111/j.1365–3156.2009.02279.x 19392741

[6] Boelaert M, Meheus F, Robays J, Lutumba P (2010) Socio-economic aspects of neglected diseases: sleeping sickness and visceral leishmaniasis. Ann Trop Med Parasitol 104: 535–542. 10.1179/136485910X12786389891641 21092391

[7] Morel CM (2006) Innovation in health and neglected diseases. Cadernos de Saúde Pública 22: 1522–1523. 10.1590/S0102–311X2006000800001 16832524

[8] Chappuis F, Sundar S, Hailu A, Ghalib H, Rijal S, et al (2007) Visceral leishmaniasis: what are the needs for diagnosis, treatment and control? Nature Reviews Microbiology 5: S7–S16. 10.1038/nrmicro1748 17938629

[9] Desjeux P (2004) Leishmaniasis: current situation and new perspectives. Comp Immunol Microbiol Infect Dis 27: 305–318. 10.1016/j.cimid.2004.03.004 15225981

[10] Guerin PJ, Olliaro P, Sundar S, Boelaert M, Croft SL, et al (2002) Visceral leishmaniasis: current status of control, diagnosis, and treatment, and a proposed research and development agenda. Lancet Infect Dis 2: 494–501. 12150849 10.1016/s1473-3099(02)00347-x

[11] Walker D, Fox‐Rushby JA (2000) Economic evaluation of communicable disease interventions in developing countries: a critical review of the published literature. Health Economics 9: 681–698. 10.1002/1099–1050(200012)9:8<681::AID-HEC545>3.0.CO;2-X 11137950

[12] Adhikari SR, Supakankunti S (2010) A cost benefit analysis of elimination of kala-azar in Indian subcontinent: an example of Nepal. J Vector Borne Dis 47: 127–139. 20834081

[13] Akhavan D (1996) Analysis of the cost-effectiveness of the leishmaniasis component of the Project for the Control of Endemic Diseases in the Northeast (PCDEN) of Brazil. Rev patol trop 25: 203–252.

[14] Anoopa Sharma D, Bern C, Varghese B, Chowdhury R, Haque R, et al (2006) The economic impact of visceral leishmaniasis on households in Bangladesh. Trop Med Int Health 11: 757–764. 10.1111/j.1365–3156.2006.01604.x 16640630

[15] Boelaert M, Lynen L, Desjeux P, Van der Stuyft P (1999) Cost-effectiveness of competing diagnostic-therapeutic strategies for visceral leishmaniasis. Bull World Health Organ 77: 667–674. 10516788 PMC2557711

[16] Das M, Banjara M, Chowdhury R, Kumar V, Rijal S, et al (2008) Visceral leishmaniasis on the Indian sub-continent: a multi-centre study of the costs of three interventions for the control of the sandfly vector, Phlebotomus argentipes. Annals of Tropical Medicine and Parasitology 102: 729–741. 10.1179/136485908X355274 19000390

[17] Griekspoor A, Sondorp E, Vos T (1999) Cost-Effectiveness Analysis of Humanitarian Relief Interventions: Visceral Leishmaniasis Treatment in the Sudan. Health Policy Plan 14: 70–76. 10351471 10.1093/heapol/14.1.70

[18] Lee BY, Bacon KM, Shah M, Kitchen SB, Connor DL, et al (2012) The economic value of a visceral leishmaniasis vaccine in bihar state, India. Am J Trop Med Hyg 86: 417–425. 10.4269/ajtmh.2012.10–0415 22403311 PMC3284356

[19] Meheus F, Balasegaram M, Olliaro P, Sundar S, Rijal S, et al (2010) Cost-effectiveness analysis of combination therapies for visceral leishmaniasis in the Indian subcontinent. PLoS Negl Trop Dis 4 Available: http://www.ncbi.nlm.nih.gov/pubmed/20838649. Accessed 8 October 2011. 10.1371/journal.pntd.0000818 PMC2935395 20838649

[20] Meheus F, Boelaert M, Baltussen R, Sundar S (2006) Costs of patient management of visceral leishmaniasis in Muzaffarpur, Bihar, India. Trop Med Int Health 11: 1715–1724. 10.1111/j.1365–3156.2006.01732.x 17054752

[21] Olliaro P, Darley S, Laxminarayan R, Sundar S (2009) Cost-effectiveness projections of single and combination therapies for visceral leishmaniasis in Bihar, India. Trop Med Int Health 14: 918–925. 10.1111/j.1365–3156.2009.02306.x 19563434

[22] Rijal S, Koirala S, Van der Stuyft P, Boelaert M (2006) The economic burden of visceral leishmaniasis for households in Nepal. Transactions of the Royal Society of Tropical Medicine and Hygiene 100: 838–841. 10.1016/j.trstmh.2005.09.017 16406035

[23] Sarnoff R, Desai J, Desjeux P, Mittal A, Topno R, et al (2010) The economic impact of visceral leishmaniasis on rural households in one endemic district of Bihar, India. Trop Med Int Health 15 Suppl 2: 42–49. 10.1111/j.1365–3156.2010.02516.x 20487423

[24] Sundar S, Gupta LB, Rastogi V, Agrawal G, Murray HW (2000) Short-course, cost-effective treatment with amphotericin B-fat emulsion cures visceral leishmaniasis. Transactions of the Royal Society of Tropical Medicine and Hygiene 94: 200–204. 10897369 10.1016/s0035-9203(00)90277-3

[25] Vanlerberghe V, Diap G, Guerin PJ, Meheus F, Gerstl S, et al (2007) Drug policy for visceral leishmaniasis: a cost-effectiveness analysis. Trop Med Int Health 12: 274–283. 10.1111/j.1365–3156.2006.01782.x 17300636

[26] Drummond MF, Sculpher MJ, Torrance GW, O’Brien BJ, Stoddart GL (2005) Methods for the Economic Evaluation of Health Care Programmes. 3rd ed Oxford University Press, USA 396 p.

[27] World Health Organization (2003) Making choices in health: WHO guide to cost-effectiveness analysis. Geneva: World Health Organization 312 p.

[28] PPP conversion factor, GDP (LCU per international $) | Data | Table (n.d.). Available: http://data.worldbank.org/indicator/PA.NUS.PPP. Accessed 30 October 2014.

[29] Husereau D, Drummond M, Petrou S, Carswell C, Moher D, et al (2013) Consolidated Health Economic Evaluation Reporting Standards (CHEERS)—Explanation and Elaboration: A Report of the ISPOR Health Economic Evaluation Publication Guidelines Good Reporting Practices Task Force. Value in Health 16: 231–250. 10.1016/j.jval.2013.02.002 23538175

[30] WHO | First WHO report on neglected tropical diseases (n.d.). WHO. Available: http://www.who.int/neglected_diseases/2010report/en/index.html. Accessed 12 April 2012.

[31] Alvar J, Yactayo S, Bern C (2006) Leishmaniasis and poverty. Trends Parasitol 22: 552–557. 10.1016/j.pt.2006.09.004 17023215

[32] Alvar J, Vélez ID, Bern C, Herrero M, Desjeux P, et al (2012) Leishmaniasis Worldwide and Global Estimates of Its Incidence. PLoS ONE 7: e35671 10.1371/journal.pone.0035671 22693548 PMC3365071

[33] Brennan A, Chick SE, Davies R (2006) A taxonomy of model structures for economic evaluation of health technologies. Health Economics 15: 1295–1310. 10.1002/hec.1148 16941543

[34] Hailu A, Musa AM, Royce C, Wasunna M (2005) Visceral leishmaniasis: new health tools are needed. PLoS Med 2: e211 10.1371/journal.pmed.0020211 16033309 PMC1181879

